# Electrochemical synthesis and corrosion protection of poly(3-aminophenylboronic acid-*co*-pyrrole) on mild steel†

**DOI:** 10.1039/d0ra07311c

**Published:** 2020-10-20

**Authors:** Hakan Sarıarslan, Erhan Karaca, Mutlu Şahin, Nuran Özçiçek Pekmez

**Affiliations:** Department of Chemistry, Hacettepe University 06800 Ankara Turkey npekmez@hacettepe.edu.tr; Department of Mathematics and Science Education, Yıldız Technical University Istanbul Turkey

## Abstract

Synthesis of poly(3-aminophenylboronic acid-*co*-pyrrole) (p(APBA-*co*-Py)) is carried out potentiodynamically on a pre-passivated mild steel (MS) surface in an oxalic acid solution containing 3-aminophenylboronic acid (APBA) and pyrrole (Py) monomers. The monomer feed ratio was determined using electrochemical impedance spectroscopy (EIS) and adhesion tests. The p(APBA-*co*-Py) coating is characterized by electrochemically and spectroscopically comparing with poly(3-aminophenylboronic acid) (p(APBA) and polypyrrole (p(Py) homopolymers. SERS, FTIR, XPS, scanning electron microscopy-wavelength dispersive X-ray and- energy dispersive X-ray spectroscopy results indicate the presence of both APBA and Py segments in the p(APBA-*co*-Py) backbone. The protective properties of the coating are investigated by Tafel and EIS measurements in a 0.50 M HCl solution. The corrosion resistance of p(APBA-*co*-Py)-coated MS (66.8 Ω cm^2^) is higher than that of p(APBA)- and p(Py)-coated, passivated, and uncoated MS. The p(APBA-*co*-Py) coating embodies the advantageous features of both homopolymers. Py units in p(APBA-*co*-Py) chains improve the protective property while APBA units carrying the –B(OH)_2_ group develop the adhesive property of the layer. EIS results show that the p(APBA-*co*-Py) coating, due to its homogeneous and compact distribution and the formation of a stable interface, enhanced corrosion resistance of MS by 87.4% for 10 hours in HCl corrosive medium.

## Abstract 2

Synthesis of poly(3-aminophenylboronic acid-*co*-pyrrole) (p(APBA-*co*-Py)) is carried out potentiodynamically on a pre-passivated mild steel (MS) surface in an oxalic acid solution containing 3-aminophenylboronic acid (APBA) and pyrrole (Py) monomers.

## Graphical abstract

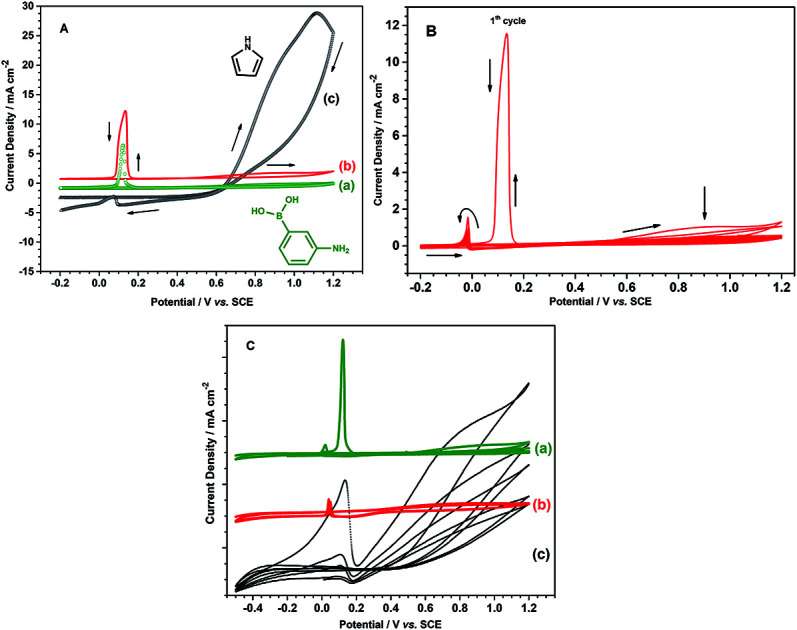

## 1. Introduction

Several techniques are available to protect metals from corrosion, such as the utilization of protective coatings (or linings), inhibitors, and cathodic or anodic protection. A conducting polymer coating on the metal surface, which is one of the active research areas in electrochemistry, acts as not only a physical but also an electronic barrier against penetration of corrosive ions.^1–5^ The electroactive character of the polymer causes the location of the reaction to shift from the metal surface to the polymer/electrolyte interface,^6^ and thus inhibits charge transfer from the corrosive solution to the metal surface or *vice versa*.^5^ In addition to this, stable interphase between polymer and metal surface forms during electrosynthesis, and thus coatings could exhibit strong adhesion and good anticorrosive properties.^7^ Among conducting polymers, polypyrrole (p(Py)) and polyaniline (PANI) have been extensively investigated in the anti-corrosion field because of the easy preparation, stability in ambient conditions, thickness controllability, and good electrical conductivity. Lacaze *et al.* obtained strongly adherent p(Py) films on pre-passivated iron and mild steel (MS) surfaces in various aqueous media containing Na_2_SO_4_, K_2_C_2_O_4_, or KNO_3_.^8^ It was shown that MS covered by the electrodeposited p(Py) films in oxalic acid yields strongly adherent and smooth polymer layers for corrosion protection.^9^ It was also reported the electrosynthesis of protective PANI films on pre-passivated MS electrode in the presence of oxalic acid, *p*-toluenesulfonic acid, and dodecylbenzene sulphonic acid.^10–15^

3-Aminophenylboronic acid (APBA), like aniline monomer, could polymerize by building chains, and unlike aniline, each unit additionally carries a –B(OH)_2_ group.^16^ Boronic acid-containing polymers with their stimuli-responsive behavior have potential applications as self-healing materials, therapeutic agents, self-regulated drug delivery systems, nucleotide adsorbents, and sensors.^17^ A few studies deal with the electrochemical synthesis of conducting polymer-based copolymer coatings such as poly(pyrrole-*co-N*-methyl pyrrole) on MS,^18^ poly(*o*-phenylenediamine) on MS,^19^ zinc modified poly(aniline-*co*-pyrrole) on low nickel stainless steel,^20^ and TiO_2_/poly(indole-*co*-aniline) on stainless steel^21^ as a protective coating against on metal corrosion. The purpose of copolymer synthesis is to obtain a coating with the advantageous features of each homopolymer. While several studies have been reported to deal with the electrochemical synthesis of poly(3-aminophenylboronic acid) (p(APBA)) and its copolymer coatings as functional sensing materials,^22–26^ their anticorrosive properties have not been studied yet.

The aim of this study is to investigate the electrosynthesis of poly(3-aminophenylboronic acid-*co*-pyrrole) (p(APBA-*co*-Py)) carrying–B(OH)_2_ group on pre-passivated MS surface as a protective coating. The p(APBA-*co*-Py) polymer was potentiodynamically synthesized in an oxalic acid solution containing APBA and Py monomers. The monomer feed ratio in the solution and electrochemical parameters were determined using electrochemical impedance spectroscopy (EIS) and adhesion tests. Characterization of p(APBA-*co*-Py) coating was carried out by cyclic voltammetry, Surface Enhanced Raman Spectroscopy (SERS), FTIR, XPS, Field Emission Scanning Electron Microscopy Energy Dispersive X-ray (FESEM-EDX) and Wavelength Dispersive X-ray (FESEM-WDX) Spectroscopy techniques by comparing with those of the p(APBA) and p(Py) homopolymers. The corrosion protection properties of p(APBA-*co*-Py) coating were investigated in highly corrosive aqueous HCl solution using Tafel and EIS techniques.

## 2. Experimentals

### 2.1. Chemicals

3-Aminophenylboronic acid (C_6_H_8_BNO_2_, Aldrich), oxalic acid (H_2_C_2_O_4_, Sigma), and hydrochloric acid (HCl, Merck) were analytical grades and used directly without any purification. Pyrrole (C_4_H_5_N, Fluka) was distilled under vacuum before the use. 3-Aminophenylboronic acid and pyrrole monomers were kept in a refrigerator under a nitrogen (Linde) atmosphere.

### 2.2. Electrochemical cell and electrodes

Electrochemical experiments were carried out with a three-electrode one compartment glass cell. We used a saturated calomel electrode (SCE) as the reference electrode, a Pt spiral as the counter electrode, and a mild steel (MS) disc electrode with a 3 mm diameter (0.07 cm^2^) as the working electrode. The main chemical composition of MS is (wt%): Fe 99.75; C 0.18; Cu 0.01; Si 0.02; P 0.02 and Mn 0.01. Before experiments, MS surface was polished with SiC abrasive paper of grit size from 400 to 2000, respectively. Following the mechanical polishing, the surface was immersed in pure water and then ethanol for 5 minutes. All electrochemical measurements were carried out at room temperature. Electrochemical experiments were performed using CH Instruments 6011D and CH Instruments 660 B.

### 2.3. Methods and instruments

Passivation of MS electrode and deposition of polymers were performed by using the potentiodynamic technique. The MS surface was passivated in the range of −0.50 V to 0.30 V at a scan rate of 4 mV s^−1^ in 0.30 M oxalic acid (Fig. S1†). The electrosynthesis of p(APBA-*co*-Py) was performed on the pre-passivated MS surface in an aqueous solution, including the monomers of Py and APBA, and the electrolyte of oxalic acid (0.1 M). In order to determine the monomer feed ratio, various Py concentrations (0.025–0.10 M) were examined while the APBA concentration was maintained at 0.10 M. And various potential intervals (the upper potential limits of 1.10 V, 1.20 V, 1.30 V, and the lower potential limits of −0.20 V, 0.20 V), scan rates (10–200 mV s^−1^), and deposition cycle numbers^10–70^ were examined to determine optimum synthesis parameters. The p(APBA) and p(Py) homopolymers were synthesized using the same procedure for comparison. The charge density applied during the synthesis of homopolymers was maintained the same as that of p(APBA-*co*-Py) (2.27C cm^−2^) The electrochemical behavior of the p(APBA-*co*-Py) and its homopolymer coatings was investigated in monomer-free 0.10 M oxalic acid in the potential region between −0.50 and 1.20 V. Tafel tests were performed by polarizing from cathodic to anodic potentials with respect to the open circuit potential at a scan rate of 1 mV s^−1^, in 0.50 M HCl solution. Before the EIS experiments, bare, passivated, and polymer-coated MS electrodes were held in 0.50 M HCl solution until they reached the steady-state open circuit potential values (*E*_OCP_). EIS tests were conducted in the frequency range of 10^5^ to 10^−2^ Hz with the amplitude of 5 mV by inputting the *E*_OCP_. Equivalent circuit models were elaborated using ZSimpWin V3.50 software (Scribner Associates Inc., UK). As the values of the equivalent circuit elements were determined, the chi-square (*χ*2) (goodness of fit) was less than 3 × 10^−3^ for each impedance spectrum.

The SERS measurements were performed using a DeltaNu Examiner Raman microscope (DeltaNu Inc., Laramie, WY, USA) with a 785 nm laser source, a motorized microscope stage sample holder, and a charge-coupled device detector. In the measurements, a 20× objective and a laser spot diameter of 3.0 μm were used. The samples were prepared by soaking the polymer-coated electrodes in a silver colloid solution of 10 μL. After drying, the SERS spectra were recorded with 140 mW laser power, for 10 s acquisition time. FTIR and XPS analyses were conducted using the Specs-Flex X-ray photoelectron spectrometer and Thermo Scientific Nicolet iS10, respectively. FESEM imaging of the polymer coatings was taken by an FEI NOVANANOSEM 650. EDX elemental mappings and WDX spectra of each coating were performed with EDAX Trident System. The amount of Fe ions released into solution from the MS specimen was determined by atomic absorption spectrometry (AA240FS, Agilent). Adherence measurements were based on the sellotape test, which is done by sticking the tape and then stripping it. Percentage adherence is determined by the ratio of the remaining film surface area to the total film surface area.^8,27,28^

## 3. Results and discussion

### 3.1. Potentiodynamic synthesis of (p(APBA-*co*-Py)) on mild steel

Prior to poly(3-aminophenylboronic acid-*co*-pyrrole) (p(APBA-*co*-Py)) synthesis, the mild steel (MS) surface was passivated to increase the stability of the substrate/polymer interphase. The passivation process was performed in the range of −0.50 V to 0.30 V at a scan rate of 4 mV s^−1^ in 0.30 M oxalic acid (Fig. S1A†). Accordingly, anodic dissolution of MS occurs in the region between −0.50 V and −0.35 V and results in the formation of Fe^2+^ ions.^18,29^ The current reaches its highest value around −0.39 V and then decreases suddenly due to the formation of a passive layer of FeC_2_O_4_·2H_2_O on the MS surface.^30^ The surface was analyzed by FTIR after being passivated in 0.3 M H_2_C_2_O_4_ to confirm the FeC_2_O_4_·2H_2_O formation (Fig. S1B†). The intense absorption bands at 1615, 1361 and 1315 cm^−1^ (*v*_(C–O)_), and 821 cm^−1^ (*δ*_(O–C–O)_ + *ν*_(C–C)_) evidence the precipitation of C_2_O_4_^−2^ on the surface, while the bands at 3334 cm^−1^ (*v*_(OH)(H_2_O)_), and 729 cm^−1^ (*δ*_(hydrated H_2_O)_) indicate the presence of hydrate water.^31^ The passive layer inhibits further dissolution of Fe without affecting the other electrochemical processes.^11,32^ It is known that the adherent and homogeneous FeC_2_O_4_ crystal layer could be deposited on the surface at very low scan rates.^33,34^

**Fig. 1:**
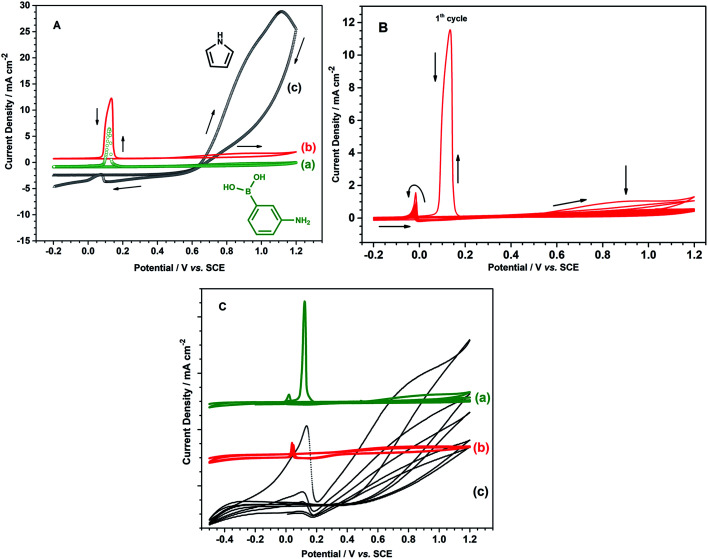
(A) First cycles obtained during electrosynthesis of (a) p(APBA) in 0.1 M APBA (b) p(APBA-*co*-Py) in 0.1 M APBA and 0.075 M Py (c) p(Py) in 0.075 M Py on the pre-passivated MS electrode, *v* = 20 mV s^−1^ (*c*_oxalic acid_ = 0.1 M as electrolyte, curves were shifted for clarity). (B) Multisweep cyclic voltammogram recorded during electrosynthesis of p(APBA-*co*-Py) on the pre-passivated MS electrode in the polymerization solution containing 0.10 M oxalic acid, 0.10 M APBA, and 0.075 M Py, *v* = 20 mV s^−1^. (C) Cyclic voltammograms obtained in monomer-free 0.1 M oxalic acid solution for (a) p(APBA), (b) p(APBA-*co*-Py), and (c) p(Py)-coated MS electrodes, *v* = 20 mV s^−1^ (curves were shifted for clarity).

The p(APBA-*co*-Py) film was deposited potentiodynamically on the pre-passivated surface from polymerization solution containing electrolyte of oxalic acid (0.10 M), and monomers of 3-aminophenylboronic acid (APBA) and pyrrole (Py). The p(APBA-*co*-Py) synthesis was performed cycling between −0.20 and 1.20 V at a scan rate of 20 mV s^−1^ with 20 cycles. Fig. 1A shows the comparison of first cycles recorded during the electrosynthesis of p(APBA-*co*-Py) and its homopolymers synthesized using the same procedure. The APBA and Py monomers begin to oxidize at about 0.60 V and 0.50 V, as seen in Fig. 1A (a) and (c), respectively. The similar oxidation potentials make it reasonable to synthesize the copolymer of two monomers.^28–30^ And the electrochemically generated radical cations of both monomers could combine to form a polymer (R1), (Fig. 1A (b)).R1
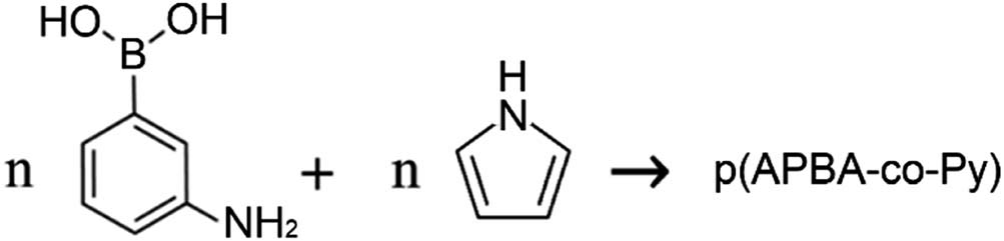


**Fig. 2:**
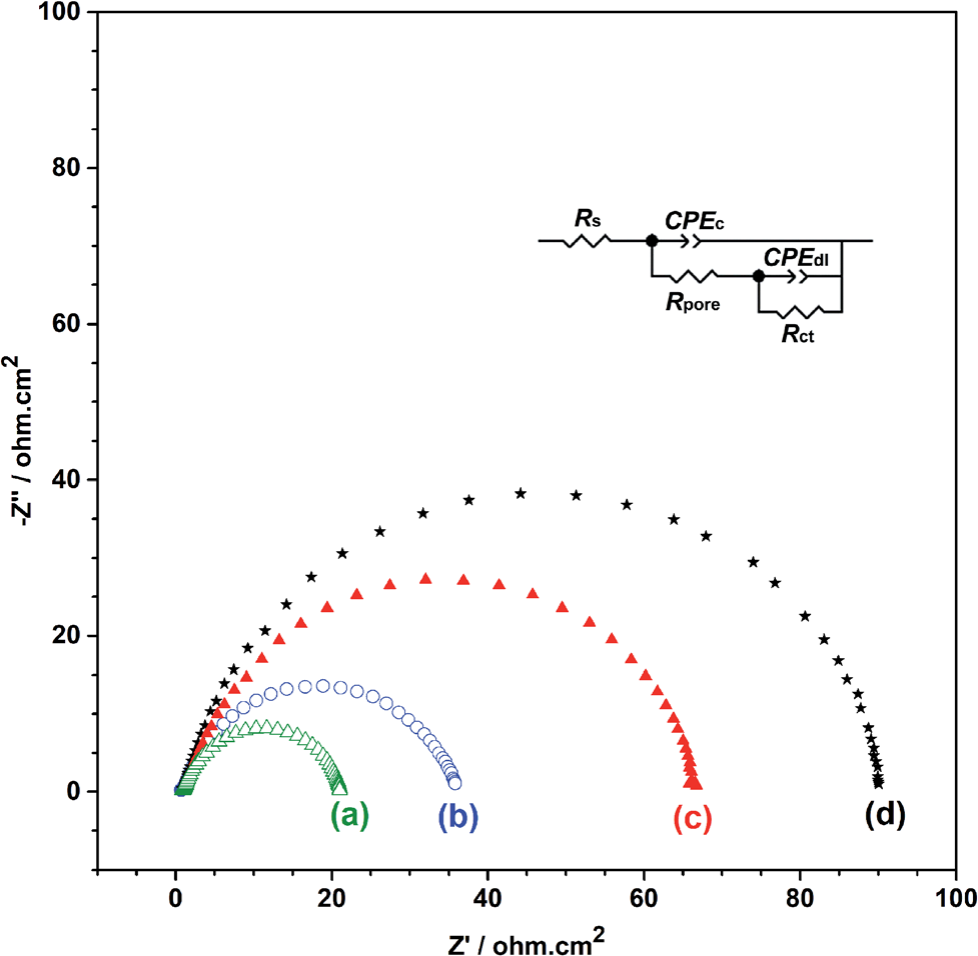
Nyquist plots obtained at *E*_OCP_ in 0.50 M HCl for the p(APBA-*co*-Py)-coated electrode prepared in the polymerization solution containing 0.10 M oxalic acid, 0.10 M APBA, and (a) 0.025 M, (b) 0.050 M, (c) 0.075 M, (d) 0.10 M Py (inset: equivalent circuit model).

In order to obtain the best quality coating for corrosion protection, the monomer feed ratio was varied. While APBA concentration was 0.1 M, various Py concentrations (0.025 M, 0.050 M, 0.075 M, and 0.10 M) were examined. The optimum Py concentration was determined by considering the polarization resistances obtained from the impedance spectra and their adherence to the surface. Fig. 2 shows the EIS spectra of the coatings measured in 0.50 M HCl. Various equivalent circuit models were examined by using ZSimpWin V 3.50 software, and the best-fitted model to the experimental data was chosen (inset of Fig. 2). This circuit model indicates the pore existence in the film and is frequently used to explain the corrosion behavior of polymer-coated metal electrodes.^33,35,36^ The circuit model includes the resistances of charge transfer (*R*_ct_), pore (*R*_pore_) and electrolyte (*R*_s_), and the constant-phase elements of coating (CPE_c_) and double-layer (CPE_dl_). In the circuit, *R*_pore_ and *R*_ct_ are serially connected. Therefore, the polarization resistance (*R*_p_) is equal to total these resistances for the polymer-coated electrode^33,35^

From the Nyquist curves shown in Fig. 2, *R*_p_ values were determined as 21.2 Ω cm^2^, 35.4 Ω cm^2^, 66.8 Ω cm^2^, and 89.5 Ω cm^2^ for the p(APBA-*co*-Py) films synthesized in the polymerization solution containing 0.025 M, 0.050 M, 0.075 M, and 0.10 M Py, respectively. As the Py concentration increases, the *R*_p_ value gradually increases, which indicates the increase of Py units in the polymer backbone. p(Py) is known to be a more protective coating than other conductive polymers in a corrosive aqueous medium.^37,38^ In addition to EIS, the adherence measurements of the p(APBA-*co*-Py) coatings were examined using the sellotape test. And, strongly adherent coatings were obtained, except for the film synthesized in the polymerization solution containing 0.10 M Py. The percentage adherence of the p(APBA-*co*-Py) synthesized in the solution containing 0.10 M Py was around 85%, while those of other p(APBA-*co*-Py) coatings were around 100%. Among the strongly adherent p(APBA-*co*-Py) coatings, the polymer obtained in the polymerization solution containing 0.075 M Py had the highest polarization resistance. Therefore, the optimum concentration of Py monomer for the p(APBA-*co*-Py) synthesis was chosen as 0.075 M.

Various potential intervals (the upper potential limits of 1.10 V, 1.20 V, 1.30 V, and the lower potential limits of −0.20 V, 0.20 V) were examined for the synthesis of p(APBA-*co*-Py) (Fig. S2†). The effect of scan rate and deposition cycle number on the polarization resistance of the coating was also investigated (Fig. S2†). The *R*_p_ values obtained from their Nyquist curves are presented in Table S1.† Accordingly, the maximum polarization resistance is obtained from the film synthesized at the potential range of −0.20 V to 1.20 V at a scan rate of 20 mV s^−1^ with 20 cycles. Fig. 1B shows the multi-sweep cyclic voltammogram recorded during electrosynthesis of p(APBA-*co*-Py) on pre-passivated MS surface using these parameters. In the first cycle, the anodic current starts to increase around 0.55 V and reaches the maximum value at 0.9 V, corresponding to the oxidation of APBA and Py monomers and the oligomers formed. This irreversible peak gradually disappears during the subsequent scans. More soluble Fe_2_(C_2_O_4_)_3_ also forms above 0.60 V and creates micropores on the surface.^14,39,40^ During the first reverse scan, a sharp oxidation peak at around 0.13 V, called repassivation peak, relates to the reconstruction of the passive FeC_2_O_4_ film within these pores.^12,41,42^ The repassivation peak shifts to more negative potentials, and its intensity decreases considerably during the subsequent cycles. This behavior indicates that the electrode surface changes after each cycle relative to the previous one due to the growing polymer. Although no obvious oxidation and reduction peaks corresponding to the synthesized polymer are observed (Fig. 1B), an adhesive and dark green colored film deposits on the surface.

**Fig. 3:**
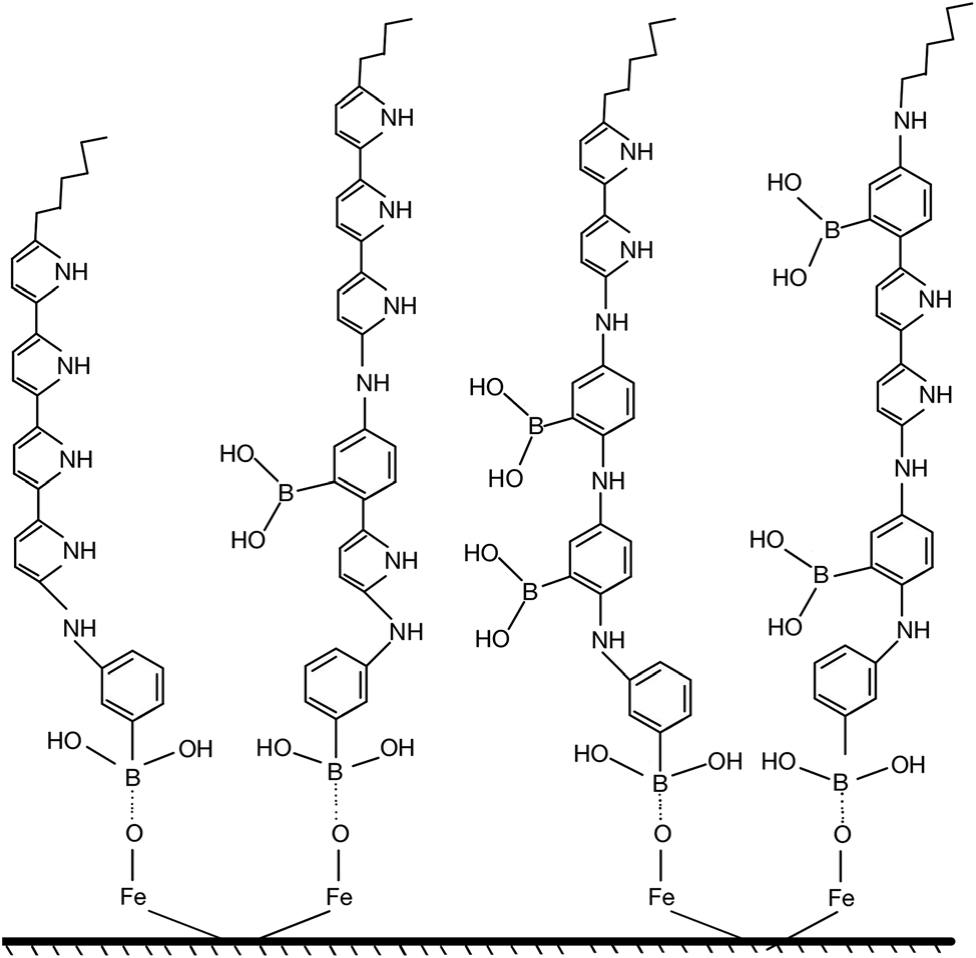
Schematic illustration of the synthesized p(APBA-*co*-Py) on the pre-passivated MS surface.

The strong adhesion of p(APBA-*co*-Py) coating might be due to the adsorption of APBA through the boronate group to iron oxides on the MS surface, as indicated in literature.^43–46^ The presence of APBA adsorption also appears in Fig. 1A. As the oxidation currents between 0.60 and 1.20 V are compared, the peak current obtained from the synthesis of p(APBA-*co*-Py) (Fig. 1A (b)) is only slightly greater than that of p(APBA) (Fig. 1A (a)). Contrarily, it was expected to be about between the currents obtained from the syntheses of p(APBA) and p(Py) (Fig. 1A (a) and (c)). It means that Py in the p(APBA-*co*-Py) synthesis solution is less oxidized, although its concentration is the same (0.075 M) as that in the p(Py) synthesis solution. As distinct from that of p(Py), Py monomer in the synthesis solution of p(APBA-*co*-Py) is oxidized on the APBA adsorbed surface. Correspondingly, it can be suggested that the polymer grows to start from the adsorbed APBA monomer. The proposed polymer structure is illustrated in Fig. 3. The APBA monomer adsorbed onto the MS surface is oxidized and combines with the cation radicals of the APBA or Py monomers formed on the electrode surface, and the growth continues like this. In other words, cation radicals of both APBA and Py monomers can be added to the growing chain due to similar oxidation potentials, as in the studies of 3-aminophenylboronic acid/3-octylthiophene,^26^ aniline/3-aminophenylboronic acid,^25^ pyrrole/bithiophene,^47^ carbazole/pyrrole,^48^ and pyrrole/1-dimethylaminopyrrole.^49^ Depending on the ratio of monomers to each other, the coatings containing the p(APBA-*co*-Py) chains with more either APBA or Py units are obtained.

The electrochemical property of p(APBA-*co*-Py) was investigated using the cyclic voltammogram obtained in monomer-free 0.10 M oxalic acid solution (Fig. 1C). Its homopolymers synthesized by the same procedure were also studied for comparison. The charge density applied during the synthesis of homopolymers was maintained the same as that of p(APBA-*co*-Py) (2.27C cm^−2^) (Fig. S3†). In the cyclic voltammogram of p(Py), broad oxidation and reduction peaks appear, indicating the formation of electroactive polymer on the surface; also, an extremely broad repassivation peak is observed in the reverse scan (Fig. 1C (c)). However, the peaks corresponding to the oxidation and reduction of p(APBA) and p(APBA-*co*-Py) polymers are almost not observed (Fig. 1C (a) and (b)). These polymers, which are not as electroactive as p(Py), exhibit almost similar electrochemical behavior except the sharp repassivation peaks. This peak is almost disappearing in the cyclic voltammogram of p(APBA-*co*-Py) coating. The difference could indicate that the deposited p(APBA-*co*-Py) polymer includes both monomer units.

### 3.2. Characterization of poly(3-aminophenylboronic acid-*co*-pyrrole)

The p(APBA-*co*-Py) film was characterized comparing with its homopolymer films using the SERS, FTIR, XPS. FESEM-WDX, FESEM imaging, and FESEM-EDX mapping analyses. The p(APBA-*co*-Py) coating was obtained on pre-passivated MS surface *via* electrosynthesis in the potential range of −0.20 V to 1.20 V at a scan rate of 20 mV s^−1^ in the polymerization solution containing 0.10 M oxalic acid, 0.10 M APBA, and 0.075 M Py.

#### 3.2.1. SERS and FTIR

**Fig. 4:**
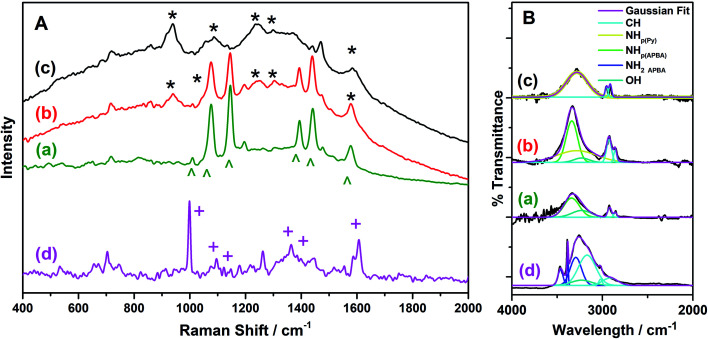
(A). SERS spectra and (B) Deconvoluted FTIR spectra of (a) p(APBA), (b) p(APBA-*co*-Py), and (c) p(Py) films synthesized on the pre-passivated MS surface, and (d) APBA monomer.

Fig. 4A illustrates the SERS spectra of the p(APBA-*co*-Py) and its homopolymer films synthesized on pre-passivated MS surface in the range of 400–2000 cm^−1^. In the spectrum of p(APBA) (Fig. 4A (a)), Raman peaks at 1578 and 1010 cm^−1^ illustrate the C–C stretching vibration of the benzene ring.^26^ The band at 1440 cm^−1^ could be attributed to both the B–O symmetric stretching and C–C stretching vibration.^26^ The peak at 1390 cm^−1^ belongs to the B–O asymmetric stretching vibration of the boronate group.^50^ The peaks at 1077 and 1146 cm^−1^ are due to the B–C stretching mode.^50^ Besides, the SERS spectrum of the monomer was taken to verify that APBA was polymerized onto the pre-passivated surface. The spectrum of APBA monomer is quite different from that of p(APBA) as seen in (Fig. 4A (a) and (d)). The C–C stretching vibration of the benzene ring is observed at 1608 and 1001 cm^−1^. B–O (also C–C) stretching and B–C stretching vibration modes respectively occur at 1363 and 1386 cm^−1^, and at 1094 and 1148 cm^−1^.^51^ Accordingly, it could be concluded the shift of the Raman bands (to 1578, 1010, 1390, 1440, 1077, and 1146 cm^−1^, respectively) belonging to p(APBA) film is due to the polymerization of monomer.^41^ In the spectrum of p(Py) (Fig. 4A (c)), the peaks at about 939 and 1242 cm^−1^ are attributed to the ring deformation.^32,52^ The broad bands appeared between 1036 and 1080 cm^−1^ is due to the C–H in-plane bending vibration.^53^ The bands at 1306 and 1583 cm^−1^ are assigned to the intercycle C–C stretching vibration mode.^26^ Thi Le *et al.* reported that the bands at 1564 cm^−1^ and 1615 cm^−1^ refers to, respectively, the fully reduced and oxidized states of p(Py); additionally, the intense peak at 939 cm^−1^ implies that the presence of partially oxidized chains.^32,50,53,54^ As seen in Fig. 4A (c), in addition to the appearance of the peak at 1583 cm^−1^ belonging to the intercycle C–C stretching vibration, the presence of the intense peak at 939 cm^−1^ indicates that p(Py) on the electrode surface is partially oxidized.^32,50,53–55^ In the SERS spectrum of p(APBA-*co*-Py) film (Fig. 4A (b)), in addition to the characteristic peaks belonging to APBA units in chains, the bands related to Py units appear at 940 and 1245 cm^−1^ (ring deformation), and 1304 cm^−1^ (intercycle C–C stretching vibration). The coexistence of these bands in the spectrum indicates the presence of both APBA and Py segments in the polymer chains.

FTIR spectra of p(APBA-*co*-Py) and its homopolymers synthesized on the pre-passivated MS electrode were recorded in the region of 4000–2000 cm^−1^. FTIR spectrum of APBA monomer was also taken to compare with that of p(APBA). Fig. 4B shows the deconvolution of these spectra. The peaks of p(Py) are observed at 3283 cm^−1^ belonging to NH stretching, and 2955 and 2900 cm^−1^ assigned to CH stretching.^51,56^ In the spectrum of p(APBA), the broad absorption band centered at 3338 cm^−1^ corresponds to the NH (of secondary amine) and OH (of -B(OH)_2_) components at 3340 and 3231 cm^−1^, respectively.^57^ The sharp peak related with aromatic CH stretching vibration is situated at 2920 and 2857 cm^−1^. On the other hand, these peaks are observed respectively at 3169 cm^−1^ (main peak), 3021 cm^−1^, and 2915 cm^−1^ in the spectrum of APBA monomer, as expected.^57^ It could be concluded that the shift towards lower wavenumber in the p(APBA) spectrum is because of the conjugation in the polymer backbone.^26,58^ Besides, the sharp peaks at 3464 and 3384 cm^−1^ (NH stretching), and 3330 cm^−1^ (overtone of the NH bending vibration) assigned to the primary amine (NH_2_) disappear in the p(APBA) spectrum.^57^ The shifting of CH peak to lower wavenumber and observing only NH of secondary amine confirm the polymerization of APBA monomer on the pre-passivated MS surface. In the case of p(APBA-*co*-Py), FTIR spectrum includes the components at 3338 cm^−1^ (NH_p(APBA)_), 3238 cm^−1^ (NH_Py_), 3231 cm^−1^ (OH), 2922 cm^−1^ (CH), and 2863 cm^−1^ (CH). As a result, the spectrum exhibits the compenents belonging to both APBA and Py segments, which indicates the formation of p(APBA-*co*-Py).

#### 3.2.2. XPS

**Fig. 5:**
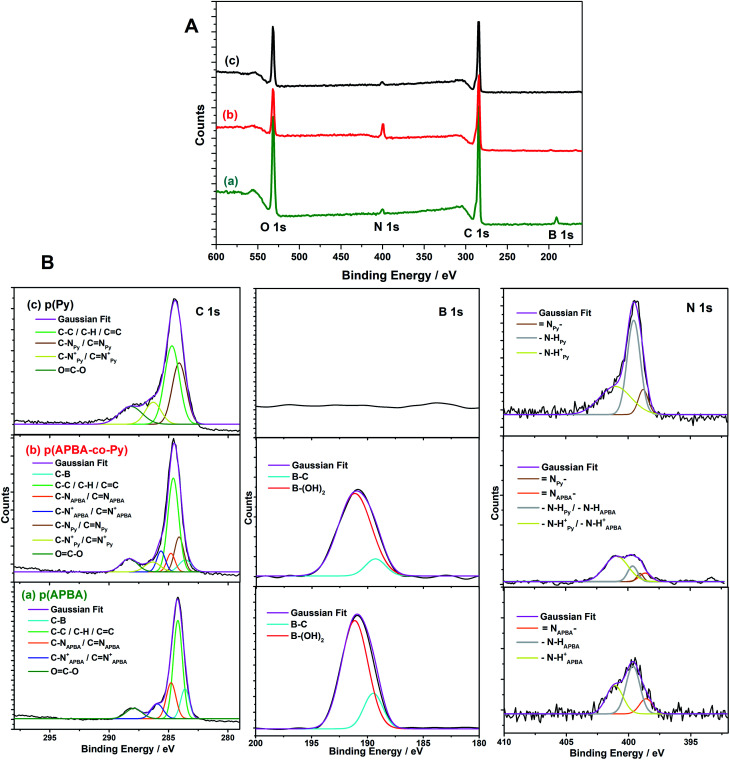
(A) Full XPS spectra and (B) Deconvoluted C 1s, B 1s, and N 1s spectra of (a) p(APBA), (b) p(APBA-*co*-Py), and (c) p(Py) films synthesized on the pre-passivated MS surface.

Fig. 5 shows the XPS spectra of the p(APBA-*co*-Py), p(APBA), and p(Py) synthesized on pre-passivated MS surface. These studies revealed the presence of C, N, and O elements in all coatings, and additionally, B element in the p(APBA) and p(APBA-*co*-Py) coatings (Fig. 5A). The presence of B 1s peak in the p(APBA-*co*-Py) spectrum indicates the presence of APBA units in the structure (Fig. 5B (b)). As B 1s peak at 191.0 eV was deconvoluted, the B–C and B–OH configurations belonging to –B(OH)_2_ group appeared.^57,59^ The high-resolution scan of C 1s and N 1s, respectively, in the ranges of 280–295 eV and 390–410 eV were deconvoluted (Fig. 5B). While the C 1s spectrum of p(Py) presents three components centered at C–C/C–H/C=C (284.2 eV), C–N/C=N (284.1 eV), and C–N^+^/C=N^+^ (286.3 eV) (Fig. 5B (c)), p(APBA) possesses same peaks centred at 284.2 eV, 284.8 eV, and 285.6 eV, respectively, and the additional peak at 283.6 eV belonging to C–B^57,60,61^ (Fig. 5B (a)). It could be deduced that the resulted polymer backbones include amine, imine, and polaron structures.^60^ In the case of p(APBA-*co*-Py), the characteristic components belonging to both APBA and Py segments are observed at individual locations (Fig. 5B (b)). This result shows the presence of both segments in the p(APBA-*co*-Py) backbone. Moreover, the O=C–O peak of oxalic acid is observed at 287.9 eV in C 1s spectra, indicating that the polymers have been doped with oxalic acid.^56^

In the high-resolution scan of N 1s of p(APBA), the peak could be deconvoluted into three components: =N– at 397.7 eV, –N–H at 399.6 eV, and –N–H^+^ at 401.0 eV (Fig. 5B (a)). The line assignments are in close agreement with the XPS results of the typical polyaniline^60^ and p(APBA).^61^ It may be attributed that the structure of the polymer contains both the imine nitrogens (=N–) of the emeraldine_p(APBA)_ state and the amine nitrogens (N–H) of the leucoemeraldine_p(APBA)_ state. Also, the identification of the C–N^+^/C=N^+^ bond belonging to polarons has confirmed the presence of oxalate dopant. In the N 1s spectrum of p(Py) (Fig. 5B (c)), –N–H and –N–H^+^ components are observed in the same location with that of p(APBA) while the peak of =N– shifts from 397.7 eV to 398.8 eV.^56,61^ The shift may be due to the nitrogen content of the pyrrole ring. In the case of p(APBA-*co*-Py) (Fig. 5B (b)), the N 1s peak broadens, and the components of APBA and Py segments appear separately. These results, which are consistent with the FTIR and SERS, imply the presence of both segments in p(APBA-*co*-Py) chains.

#### 3.2.3. FESEM-WDX analysis

**Fig. 6:**
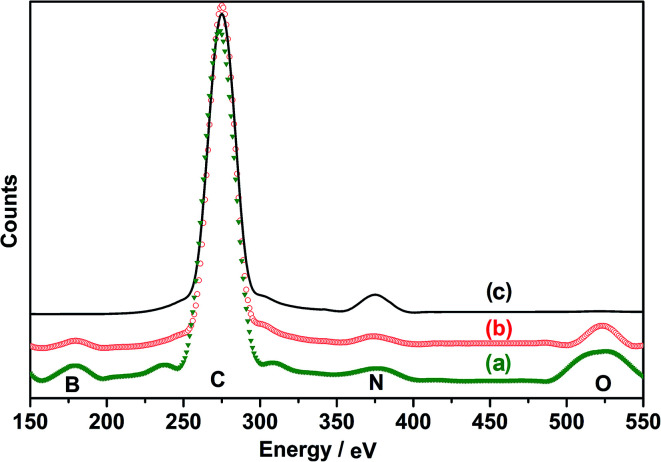
WDX spectra of (a) p(APBA), (b) p(APBA-*co*-Py), and (c) p(Py) films synthesized on the pre-passivated MS surface.

In order to confirm the presence of both Py and APBA units in the p(APBA-*co*-Py) chains, its FESEM-WDX analysis^62,63^ was performed together with those of homopolymers (Fig. 6). The weight percentage of each element in polymer coatings was obtained from the area under the peak, and their atomic percentages were calculated (Table S1†). While C-element originates from both polymers and C_2_O_4_^−2^ dopant, the N– and B– elements come only from the polymers. The atomic ratio of N/B is 0.93 and 1.9 for the p(APBA) and p(APBA-*co*-Py) film, respectively (Table S2†). The difference between these two ratios should be the relative percentage of APBA and Py units in the resulting coating. It can be concluded that the relative percentage of the APBA and Py units is 1 : 1 in the p(APBA-*co*-Py) chains.

#### 3.2.4. FESEM imaging and FESEM-EDX mapping

**Fig. 7:**
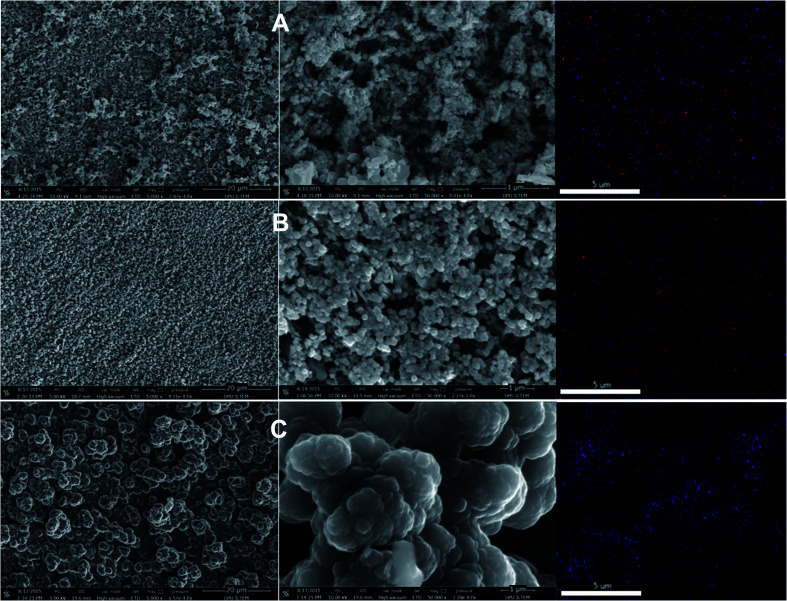
SEM images at 2 μm and 20 μm scales, and elemental mappings of (A) p(APBA) (B) p(APBA-*co*-Py) and (C) p(Py) coatings deposited on the pre-passivated MS surface.

FESEM images of the p(APBA-*co*-Py), p(APBA), and p(Py) coatings deposited on the pre-passivated MS electrode were presented in Fig. 7. The p(APBA) film (Fig. 7A) exhibits a granular structure, while the p(Py) film (Fig. 7C) has a cauliflower-like structure.^33,48,64^ On the other hand, p(APBA-*co*-Py) film exhibits a globular structure of different sizes, generally between 100 and 200 nm, resembling cauliflower appearance (Fig. 7B). Besides, the p(APBA-*co*-Py) coating has a more smooth, compact, and homogeneous surface than the others. Additionally, N- and/or B-EDX mapping analysis was performed to investigate the spatial distribution of each coating (Fig. 7). Accordingly, a homogenous distribution was observed in the N-EDX mapping of p(Py) coating, and the B- and N-EDX mappings of p(APBA) coating. B- and N-elements in also mapping of p(APBA-*co*-Py) coating distribute uniformly.

### 3.3. Corrosion protection of p(APBA-*co*-Py) coating

Corrosion performance of the p(APBA-*co*-Py) coating was investigated comparing with its homopolymer films using the Tafel and EIS analyses. The p(APBA-*co*-Py) synthesis was carried out on pre-passivated MS surface in the potential range of −0.20 V to 1.20 V at a scan rate of 20 mV s^−1^ in the polymerization solution containing 0.10 M oxalic acid, 0.10 M APBA, and 0.075 M Py. The p(APBA) and p(Py) homopolymers were synthesized using the same procedure with p(APBA-*co*-Py) (Fig. S3†). The charge density applied during the synthesis of homopolymers was maintained the same as that of p(APBA-*co*-Py) (2.27 C cm^−2^).

#### 3.3.1. Tafel test

**Fig. 8:**
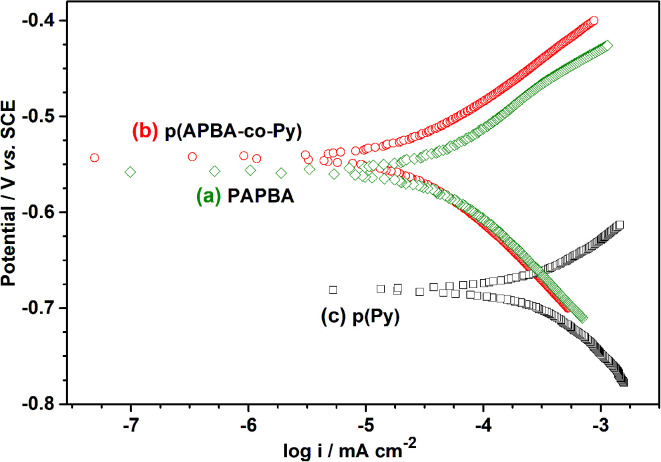
Tafel polarization curves recorded in 0.5 M HCl for (a) p(APBA), (b) p(APBA-*co*-PPy), and (c) p(Py) coatings on the pre-passivated MS electrodes.

Tafel analysis may be used to compare the protective properties of electroactive polymer coatings, although it is usually insufficient alone.^33^Fig. 8 shows Tafel curves of p(APBA-*co*-Py), p(APBA), and p(Py)-coated MS electrodes in 0.50 M HCl as a corrosive medium. The values of corrosion potential (*E*_corr_) and corrosion current density (*i*_corr_) were obtained by the extrapolation of the linear portions of Tafel plots. The *i*_corr_ values decreased while *E*_corr_ values shifted toward to the nobler direction in the order of p(Py) (6.2 μA cm^−2^, −0.68 V), p(APBA) (0.7 μA cm^−2^, −0.56 V), and p(APBA-*co*-Py) (0.5 μA cm^−2^, −0.54 V). After all, the p(APBA-*co*-Py)-coated electrode has the lowest *i*_corr_ and the noblest *E*_corr_, *i.e.*, p(APBA-*co*-Py) is the coating that most restricts the anodic and cathodic reactions of MS in the aggressive medium. On the other hand, the p(Py) coating has not provided smaller *i*_corr_ and nobler *E*_corr_. It could be because p(Py) is the most electroactive polymer or has the highest doping level among the polymers synthesized. To sum up, not only the corrosion of MS but also the redox process of the electroactive polymer may be contributed to the *i*_corr_ values.^65,66^

#### 3.3.2. EIS test

**Fig. 9:**
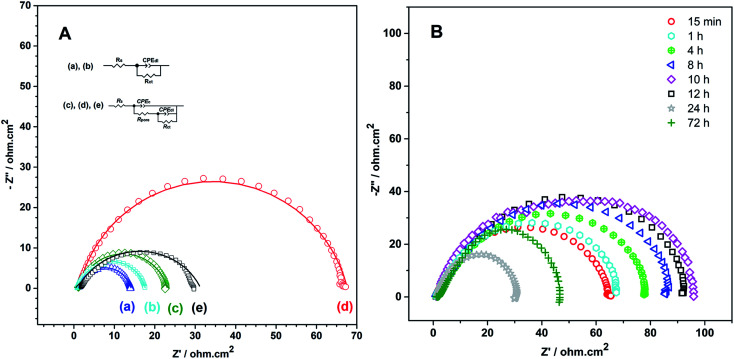
(A). Nyquist plots recorded at *E*_OCP_ in 0.50 M HCl for (a) bare and (b) passivated MS electrodes, and (c) p(APBA), (d) p(APBA-*co*-Py), and (e) p(Py) coatings on the pre-passivated MS electrodes. (inset: equivalent circuit models; solid lines and points represent fitted and experimental data, respectively). (B) Nyquist plots recorded during immersion time of 72 hours of p(APBA-*co*-Py)-coated electrode in 0.50 M HCl at *E*_OCP_.

Impedance spectra of the bare, passivated, and homopolymer-coated MS electrodes were conducted in 0.50 M HCl and compared to that of the p(APBA-*co*-Py)-coated electrode (Fig. 9A) (Table 1). The equivalent circuits used for modeling of coated and uncoated MS electrodes were presented in the inset of Fig. 9A. The fittings obtained from the equivalent circuits are shown in Fig. 9A as solid lines. Accordingly, the proposed models are a good representation of the phenomena which may occur in both the high- and low-frequency parts of the spectra. The parameters obtained where the chi-square (*χ*^2^) values are less than 6 × 10^−3^ are listed in Table 2. In addition to the parameters, the protection efficiency (PE%) of the polymer-coated electrodes was computed using the following equation^33^ and given in Table 2.

1 PE% = (*R*_p_ − *R*_p,0_/*R*_p_) × 100

*R*_p,0_ and *R*_p_ is the polarization resistance of the bare and polymer-coated electrodes, respectively. As seen in Table 2, *R*_p_ (and PE%) values of the p(APBA) and p(Py) homopolymer coatings are respectively 22.2 and 28.4 Ω cm^2^ (41.3% and 54.2%) while that of p(APBA-*co*-Py) coating is 66.8 Ω cm^2^ (80.5%). Accordingly, p(APBA-*co*-Py) coating exhibits the maximum polarization resistance similar to Tafel test results. Moreover, its *R*_p_ value is also higher about five times than that of the uncoated MS electrode, as seen in Table 2. High *R*_p_ value is associated with the effective corrosion resistance, which is proof of the formation of a stable polymer layer and a passive layer on the electrode/polymer interface.^5^ The adherence of p(APBA) and p(Py) coatings were also examined and were found as about 100% and 25%, respectively. The ability of p(APBA-*co*-Py) coating to adhere to the steel surface (100%) is better than p(Py) coating. Consequently, p(APBA-*co*-Py) coating has both the highest *R*_p_ (and PE%) value and better adhesive property than p(Py), *i.e.*, the coating embodies the advantageous features of both homopolymers. Py segments in p(APBA-*co*-Py) chains improve the electronic barrier property of the coating against penetration of corrosive ion, while APBA segments carrying –B(OH)_2_ group develop its adhesive property. As a result, its protective property is better than those of homopolymer coatings.

**Table 1:** Impedance parameters obtained by fitting the impedance spectra given in Fig. 9 for the electrodes

Electrode	*R*_s_/Ω cm^2^	CPE_c_/μF^n1^ cm^−2^	*R*_pore_/Ω cm^2^	CPE_dl_/μF^n2^ cm^−2^	*R*_ct_/Ω cm^2^	*R*_p_/Ω cm^2^	PE/%
MS	1.1	—	—	407	13.0	13.0	—
Passivated MS	1.0	—	—	391	16.3	16.3	20.2
p(APBA)	0.9	14.9	0.5	360	21.7	22.2	41.3
p(APBA-*co*-Py)	0.9	48.4	0.8	71.0	66.0	66.8	80.5
p(Py)	0.4	116	1.5	537	26.9	28.4	54.2

**Table 2:** Impedance parameters obtained by fitting the impedance spectra for p(APBA-*co*-Py) coating on the pre-passivated MS electrode immersed in 0.5 M HCl for 72 h

Immersion time/h	*R*_s_/Ω cm^2^	CPE_c_/μF^n1^ cm^−2^	*R*_pore_/Ω cm^2^	CPE_dl_/μF^n2^ cm^−2^	*R*_ct_/Ω cm^2^	*R*_CE_/Ω cm^2^	PE/%
0.25	0.9	48.4	0.8	71.0	65.6	66.4	80.4
1	0.9	237	3.5	106	81.4	81.9	84.7
4	1.0	169	4.2	124	89.2	93.6	85.5
8	1.0	19.1	11.3	31.6	90.3	102	87.2
10	1.0	259	11.9	36.0	91.7	104	87.4
12	0.9	75.6	0.7	327	72.7	73.4	82.3
24	1.9	112	0.6	293	43.4	44.0	70.4
72	1.4	2.90	0.4	1471	30.2	30.6	57.4

The impedance response of p(APBA-*co*-Py)-coated MS electrode has been investigated for 72 hours after immersed in 0.50 M HCl (Fig. 9B). As seen in Table 2, the *R*_p_ value gradually increases for 10 hours and then decreases; after 72 hours, the *R*_p_ value is still greater than that of the bare electrode. Similar to the behavior of polyaniline and its derivatives but unlike p(Py),^33,35^ this trend may be due to redox processes of small APBA segments in the p(APBA-*co*-Py) structure. The role of APBA segments in the p(APBA-*co*-Py) chains in protecting the MS surface from corrosion could be expressed as following (R2), (R3):^33^

R2 2Fe + 3EM_p(APBA)_^2+^ + 3H_2_O → Fe_2_O_3_ + 3LE_p(APBA)_ + 6H^+^

R3 LE_p(APBA)_ + ½O_2_ + H_2_O → EM_p(APBA)_^2+^ + 2OH^−^

The emeraldine form of APBA segment (EM_p(APBA)_^2+^) is reduced to its leucoemeraldine form (LE_p(APBA)_) as the immersion time increases.^67,68^ Concurrently, Fe_2_O_3_ occurs in the pores of polymer coating and/or between polymer/MS interface.^64,69,70^ Polymer coating restricts the diffusion of the corrosion products from MS; thus, corrosion products could protect by covering the steel surface. As the ratio of reduction increases, the reduced film (LE_p(APBA)_) is oxidized to EM_p(APBA)_^2+^ again by oxygen in solution (R3). But, the reaction rate of (R3) should be slower than that of (R2), which is why the *R*_p_ value begins to decrease after 10 hours. Eventually, oxalate ions exchange with chloride ions. After the prolonged exposure time to the corrosive medium, the penetration of corrosive chloride ions on the surface can not be hindered, and the coating loses its protective behavior. In order to confirm the presence of Fe_2_O_3_ formed on the p(APBA-*co*-Py)-coated surface as a result of corrosion, the polymer coating was scraped after being immersed in 0.5 M HCl, and XRD analysis of the surface was performed (Fig. S4†). Accordingly, α-Fe_2_O_3_ related peaks are observed even though the intensity of the peaks is low due to the scraping of the coating from the surface. Besides, the amount of Fe ions released into solution from the coated and uncoated MS specimen at different immersion times in 0.5 M HCl was analyzed by atomic absorption spectrometry. The Fe ion contents were 12.9, 19.1, and 26.1 mg cm^−2^ h^−1^ after respectively 24, 48, and 72 h for bare MS, while they were 4.1, 6.35, and 18.6 mg cm^−2^ h^−1^ for the coated MS specimen. It could be concluded that the p(APBA-*co*-Py) coating could protect the surface longer by reducing the dissolution of MS.

Table S3† exhibits the comparison of the protective properties of p(APBA-*co*-Py) coating with those of the conductive polymer-based coatings on MS in the literature. Their protection efficiencies vary between 70% and 99%, and the result obtained in this study is within this range (80.5%). The p(APBA-*co*-Py) coating, due to its chemical and physical properties and the formation of a stable interface, effectively protected the mild steel against corrosion up to 10 hours in a highly acidic medium. The coating exhibits the properties of good adherence to the surface due to the APBA units and a good electronic barrier (*i.e.*, protection efficiency) against penetration of corrosive ion due to the Py units. Consequently, the p(APBA-*co*-Py) coating could represent a promising material in corrosion protection application.

## 4. Conclusions

The electrochemical synthesis of the p(APBA-*co*-Py) film on pre-passivated mild steel electrode was performed by using the potentiodynamic technique in the oxalic acid (0.1 M) solution containing APBA (0.1 M) and Py (0.25–0.1 M) monomers. The polarization resistance of p(APBA-*co*-Py) coating gradually increased with the increase of the concentration of Py monomer in the polymerization solution; on the other hand, the adhesive property decreased after 0.75 M Py. Therefore, the synthesis of p(APBA-*co*-Py) was carried out in the presence of 0.075 M Py. The p(APBA-*co*-Py) film has strong adherent properties that may be due to the complexation of –B(OH)_2_ group with FeO_*x*_ at the polymer/electrode interface. The spectroscopic results indicate the presence of both APBA and Py segments in the p(APBA-*co*-Py) backbone. While a homogenous distribution is observed in B- and N-EDX mappings of p(APBA-*co*-Py) coating, it is exhibited from FESEM-WDX analysis that the relative percentage of the APBA and Py units is 1 : 1. The corrosion protection performance of the p(APBA-*co*-Py) coating was investigated by the EIS and Tafel tests in 0.50 M HCl. In comparison with the homopolymer coatings synthesized using the same procedure, p(APBA-*co*-Py) exhibited the smallest *i*_corr_ (and the noblest *E*_corr_) value. Similarly, it had the highest polarization resistance obtained in EIS measurements, and its *R*_p_ value was also higher about five times in comparison with the bare electrode. The p(APBA-*co*-Py) coating, due to its homogeneous and compact distribution and the formation of a stable interface, effectively protected the mild steel against corrosion up to 10 hours in highly acidic medium.

## Conflicts of interest

There are no conflicts to declare.

## Supplementary Material

RA-010-D0RA07311C-s001
