# Supplementary material for: Electrochemical synthesis and corrosion protection of poly(3-aminophenylboronic acid-co-pyrrole) on mild steel
Source: RSC Adv. 2020 Oct 20;10(63):38548–60. doi: 10.1039/d0ra07311c (PMC9057268; doi:10.1039/d0ra07311c)
Supplement: RA-010-D0RA07311C-s001 [file RA-010-D0RA07311C-s001.pdf]

Electronic Supplementary Material (ESI) for RSC Advances.
This journal is © The Royal Society of Chemistry 2020

Supplementary Data
Electrochemical synthesis and corrosion protection of poly(3-aminophenylboronic acid-co-pyrrole) on mild steel

Hakan Sarıarslan[a], Erhan Karaca[a], Mutlu Şahin[b], Nuran Özçiçek Pekmez [a]*

[a]Hacettepe University, Department of Chemistry, 06800 Ankara, Turkey
[b]Yıldız Technical University, Department of Mathematics and Science Education, Istanbul, Turkey

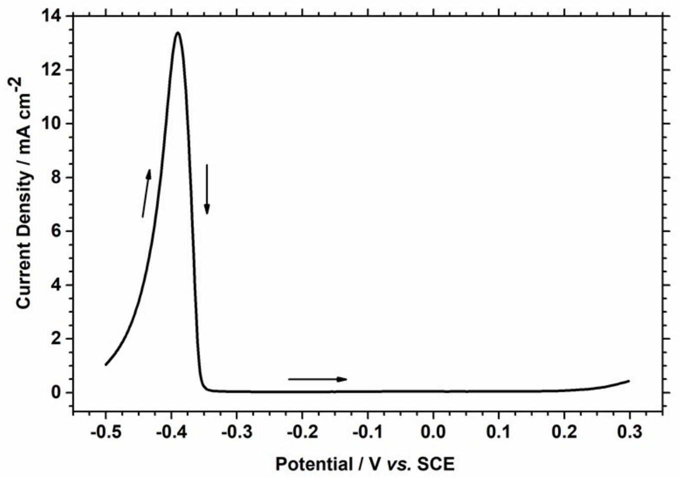

Figure S1A. Passivation of mild steel electrode in 0.30 M oxalic acid in the potential range of −0.50 to 0.30 V, $v = 4$ mV s^{-1}.

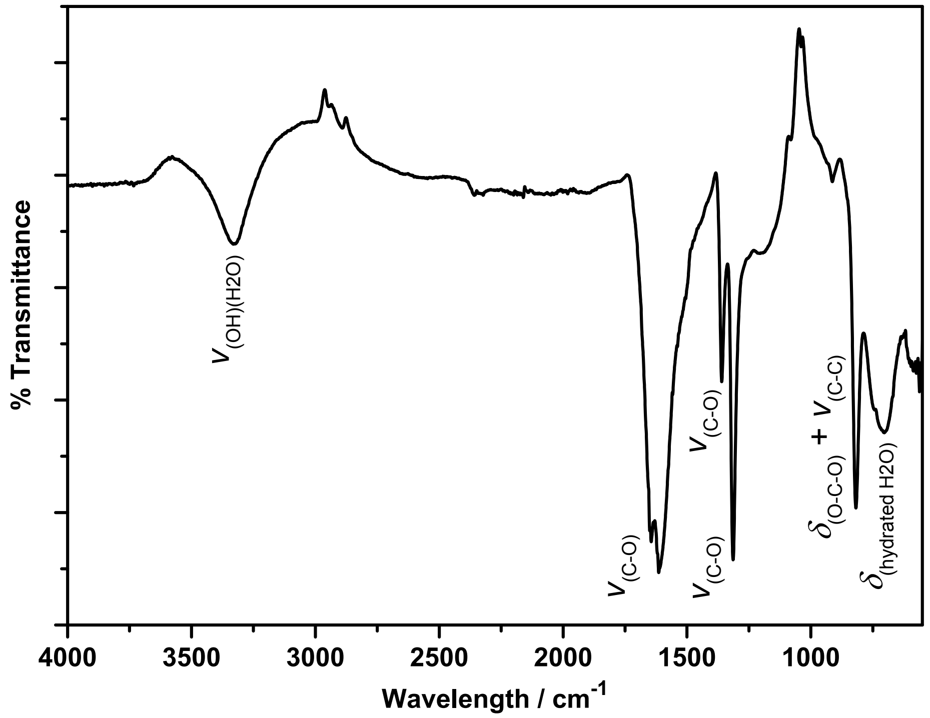

Figure S1B. FTIR spectrum of the mild steel surface after being passivated in 0.3 M $H_2C_2O_4$

Optimization of potentiometric parameters in the p(APBA-co-Py) synthesis

Various potential intervals (the upper potential limits of 1.10 V, 1.20 V, 1.30 V, and the lower potential limits of −0.20 V, 0.20 V) were examined for the p(APBA-co-Py) synthesis on the pre-passivated MS surface. The R_p values obtained from their Nyquist curves (Fig. S2A) are presented in Table S1. Accordingly, the maximum polarization resistance is obtained from the film synthesized at the potential range of −0.20 V to 1.20 V. The polymer film could be incurred over-oxidation as the upper potential limit is a more positive potential than 1.20 V [1-3]. On the other hand, the generation of a sufficient amount of radical cations could not be realized at the upper potential of 1.10 V. Thus, the adequate amount of film could not be deposited. As the lower potential limit, -0.20 V is more appropriate than 0.2 V. It could be due to the fact that the passive oxide layer on the surface needs to be renewed at around 0.12 V (repassivation peak, see Fig.1B for details) in each reverse scan during the electropolymerization.

In order to elucidate the effect of scan rate, the p(APBA-co-Py) films were obtained via electrosynthesis at various scan rates between 10 mV $s^{-1}$and 200 mV s^{-1} at the potential range of −0.20 V to 1.20 V. The R_p values obtained from their Nyquist curves (Fig.S2B) are presented in Table S1. The highest polarization resistance is obtained as the film is deposited at a scan rate of 20 mV s^{-1}. The polymer film might be incurred over-oxidation at a scan rate of 10 mV s^{-1} [1-3]. At higher scan rates, the time for the generation of a sufficient amount of the monomer radical cations may be short.

The effect of the deposition cycle number on the polarization resistance of the p(APBA-co-Py) coating was investigated (Fig.S2C). As seen in Table S1, the highest polarization resistance is obtained as the film is deposited with 20 cycles. At 10 cycles, not enough polymer could be synthesized to cover the MS surface completely. On the other hand, above the 20 cycles, the polymer film close to the surface might be incurred over-oxidation [1-3].

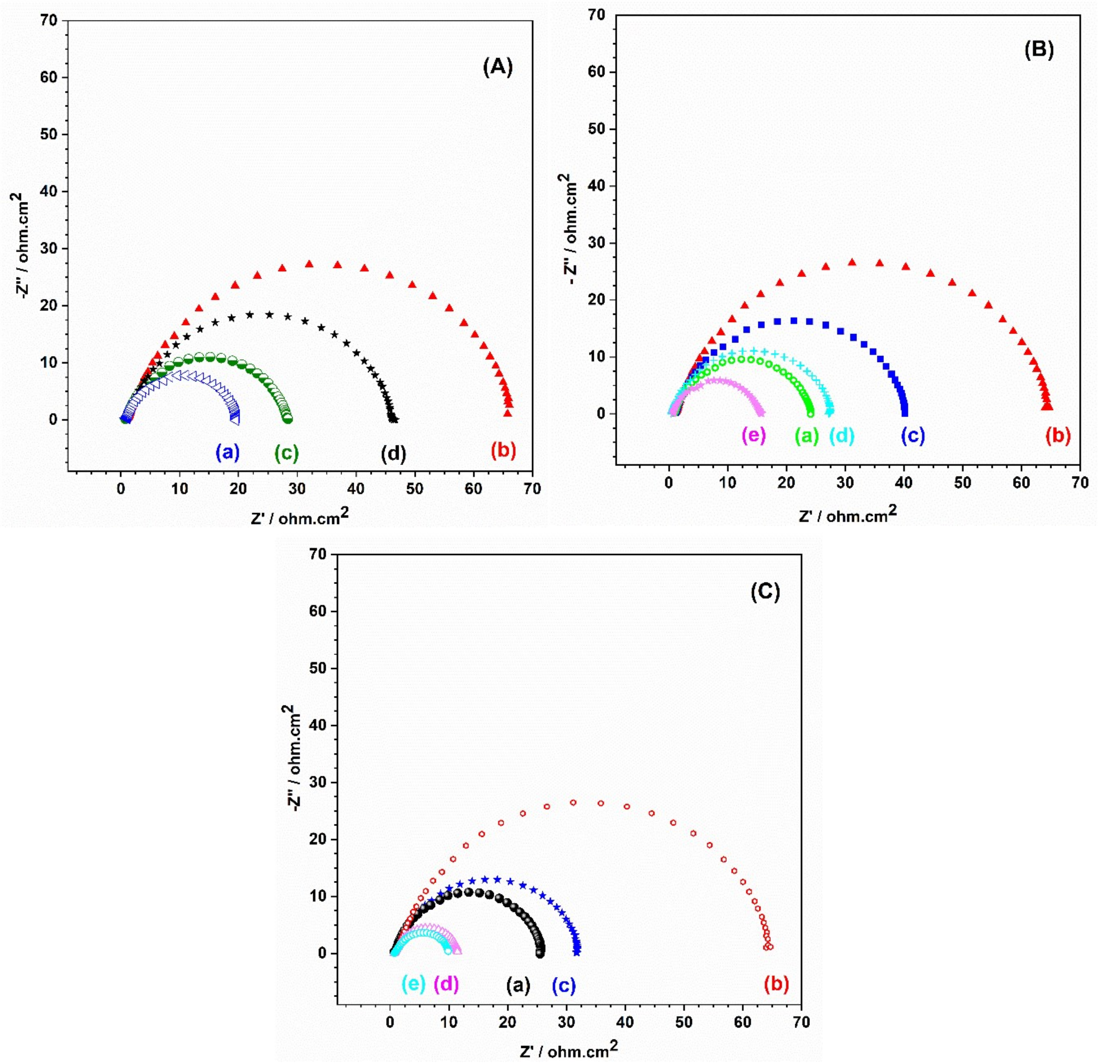

Figure S2. Nyquist plots obtained at E_{OCP} in 0.50 M HCl for p(APBA-co-Py) coated electrode obtained in the polymerization solution containing 0.10 M oxalic acid, 0.10 M ABPA, and 0.075 M Py **(A)** with 20 cycles at a scan rate of 20 mV s^{-1} in the potential region between −0.20 V and **(a)** 1.10 V **(b)** 1.20 V **(c)** 1.30 V, and (d) between 0.20 V and 1.20 V, **(B)** with 20 cycles in the potential region between −0.20 V and 1.20 V at a scan rate of **(a)** 10 mV s^{-1} **(b)** 20 mV s^{-1} **(c)** 50 mV s^{-1} **(d)** 100 mV s^{-1} **(e)** 200 mV s^{-1} **(C)** in the potential region between −0.20 V and 1.20 V at a scan rate of 20 mV s^{-1} with **(a)** 10 **(b)** 20 **(c)** 30, **(d)** 50, **(e)** 70 cycles.

Table S1. Optimization of potentiodynamic parameters from the Nyquist curves (given in Fig.S2) for the p(APBA-co-Py) synthesis on the pre-passivated MS electrode.

	**R_p / Ω cm^2**
Potential range **/ V**	
(−0.20) – (1.10)	18.9
(−0.20) – (1.20)	66.8
(−0.20) – (1.30)	27.6
(0.20) – (1.20)	45.3
Scan rate **/ mV s^{-1}**	
10	23.9
20	66.8
50	40.2
100	28.3
200	15.5
Cycle number	
10	26.2
20	66.8
30	32.8
50	11.1
70	9.50

Synthesis of p(APBA) and p(Py) homopolymers

The characterization and corrosion performance of p(APBA-co-Py) coating was investigated comparing with its homopolymer films. The p(APBA) and p(Py) homopolymers were synthesized using the same procedure with p(APBA-co-Py) (in the potential range of−0.20 V to 1.20 V at a scan rate of 20 mV s^{-1}). The charge density applied during the synthesis of homopolymers was maintained the same as that of p(APBA-co-Py) (2.27 C cm^{-2}). According to the multi-sweep cyclic voltammogram recorded during the polymerization of Py (Fig. S3 (b)), the oxidation peak of Py observed above 0.60 V gradually disappears while broad oxidation and reduction peaks of p(Py) appear at more negative potentials. The broad peaks increase in intensity as the film grows. After three cycles, a black film is observed on the electrode surface. In the case of p(APBA) (Fig.S3 (a), the electrooxidation and reduction characteristics are almost similar to p(APBA-co-Py) (Fig.1B), except their peak intensities.

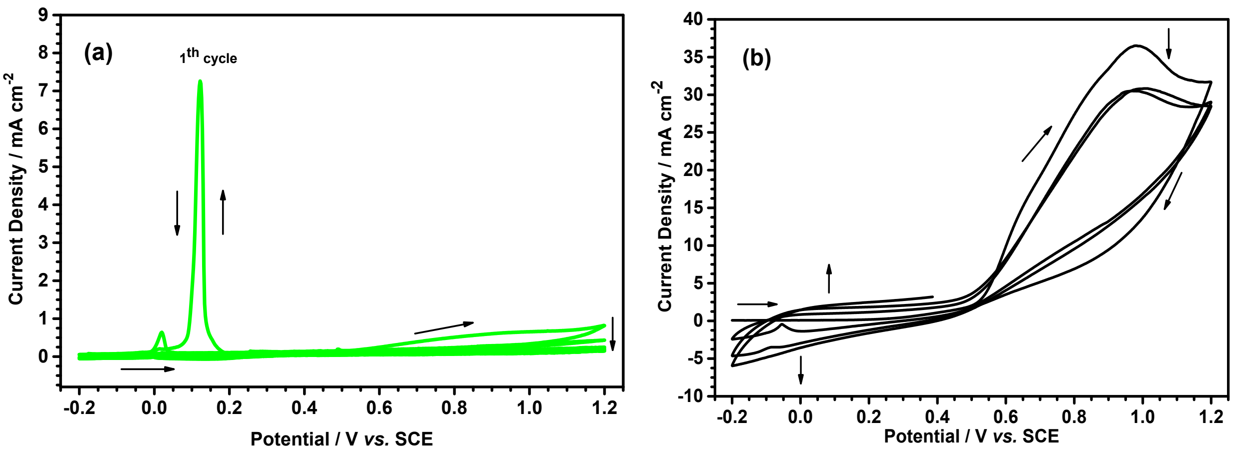

Figure S3. Potentiodynamic curve recorded during electrosynthesis of **(a)** p(APBA) in the oxalic acid solution containing 0.10 M APBA **(b)** p(Py) in the oxalic acid solution containing 0.075 M Py on pre-passivated MS electrode in the potential region between -0.20 V and 1.20 V with 20 cycles, $v = 20$ mV s^{-1} ($c_{\text{oxalic acid}} = 0.10$ M).

Table S2. Results of WDX analysis for the coated electrodes

Atomic ratio / %	p(Py)	p(APBA)	p(APBA-co-Py)
B	---	4.1	2.1
C	95.4	82.3	88.7
N	4.6	3.8	4.0
O	---	9.9	5.1

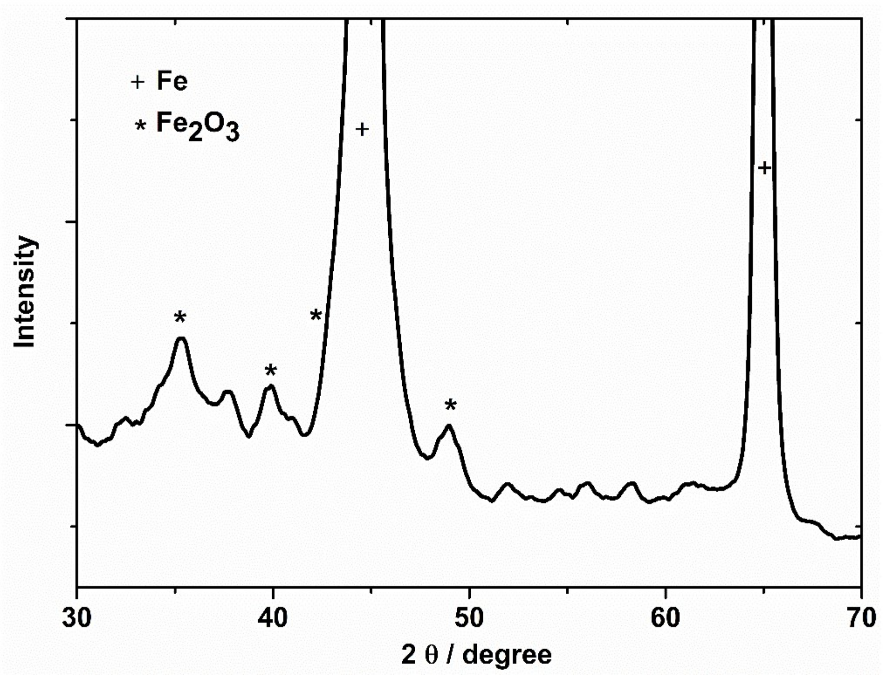

Figure S4. XRD spectrum taken from the coating/MS interface after scraping the p(APBA-co-Py) coating immersed in the 0.5 M HCl solution. (Fe (JCPDS 85-1410): 44.8°, 65.0° and α–Fe_2O_3 (JCPDS 85-0599): 35.2°, 39.8°, 42.8°, 48.9°)

Table S3. Protective property of the conductive polymer-based coatings on MS in the literature.

Coating	*Electrolyte*	R_p / Ω cm^2	PE / %	Ref.
Drilled epoxy/PANi	0.1 M HCl			
Poly(o-phenylenediamine)-H_3PO_4	3.5% NaCl	1761	80.52	[4]
Polypyrrole/PANi	0.1 M NaCl	55.4		[5]
PANi/Chitosan	0.5 M HCl	61.6		[6]
PPy/SnO_2	0.5 M H_2SO_4	30		[7]
PPy/CeO_2	0.5 M H_2SO_4	34.9		[7]
PPy/TiO_2	0.5 M H_2SO_4	34.4		[7]
PPy/ZnO	0.5 M H_2SO_4	59.9		[7]
PPy/Al_2O_3	0.5 M H_2SO_4	154		[5]
PPy/dodecylbenzenesulfonate	0.5 M HCl	402	99.7	[8]
p(aniline-co-N-ethylaniline)	0.1 M HCl	281	85.1	[9]
p(aniline-co-o-anisidine)	3% NaCl	22700	92.7	[10]
p(m-phenylendiamin-co-o-aminophenol)	0.5 M HCl	842	97.5	[11]
p(pyrrole-co-o-toluidine)	0.1 M HCl	734	97.3	[12]
p(N-methylaniline)/PPy	0.5 M HCl	26310	70.1	[13]
p(APBA-co -Py)	0.5 M HCl	66.8	80.5	This study

[1] N.C.T. Martins, T. Moura e Silva, M.F. Montemor, J.C.S. Fernandes, M.G.S. Ferreira, Electrochim. Acta, 53 (2008) 4754-4763.

[2] F.Ç. Cebeci, H. Geyik, E. Sezer, A. Sezai Sarac, J. Electroanal. Chem., 610 (2007) 113-121.

[3] N. Pekmez, K. Pekmez, A. Yıldız, J. Electroanal. Chem., 370 (1994) 223-229.

[4] J.-W. Zhang, Y. Li, Y.-S. Ding, K.-F. Pan, Q. Zhao, Jom, (2019).

[5] F. Yildirim, Asian J. Chem., 31 (2019) 1532-1538.

[6] P. Kong, H. Feng, N. Chen, Y. Lu, S. Li, P. Wang, RSC Advances, 9 (2019) 9211-9217.

[7] R. Babaei-Sati, J. Basiri Parsa, M. Vakili-Azghandi, Synth. Met., 247 (2019) 183-190.

[8] B. Zeybek, Ş. Yüksel, J. Adhes. Sci. Technol., 32 (2018) 2464-2480.

[9] M. Mobin, J. Aslam, R. Alam, Arabian Journal for Science and Engineering, 42 (2016) 209-224.

[10] P.M. Raotole, P. Koinkar, B. Joshi, S.R. Patil, J. Coat. Technol. Res., 12 (2015) 757-766.

[11] A. Madhankumar, N. Rajendran, Prog. Org. Coat., 76 (2013) 1445-1453.

[12] M. Mobin, N. Tanveer, Protection of Metals and Physical Chemistry of Surfaces, 48 (2012) 243-250.

[13] B. Zeybek, N. Özçiçek Pekmez, E. Kılıç, Electrochim. Acta, 56 (2011) 9277-9286.
